# Cases of Laryngismus Stridulus

**Published:** 1858-07

**Authors:** Jno. C. K. Crooks

**Affiliations:** Honeoye, Ontario Co., N. Y.


ART. VI.—*Cases of Laryngismus Stridulus.* By JNO. C. K. CROOKS, M. D., Honeoye, Ontario Co., N. Y.

I take the liberty of sending you the history of two cases of "Laryngismus Stridulus," occurring in adult females; which, should you deem of sufficient interest, please give a place in your valuable Journal. One of said cases came under my own observation, while the other was kindly furnished me by a medical friend.

CASE I. Mrs. P., aged thirty-eight; is married, and the mother of four children; has had ill health since the birth of her last child, which, at the time of the attack, was three years old. She recovered slowly from her confinement — was a good deal prostrated — had pain in the back, leucorrhœa, "bearing down," tenderness over the hypogastrium, painful micturition, &c., to which *uterine* symptoms, was added severe and prolonged "nursing sore mouth."

The stomatitis finally yielded to remedies, but the symptoms of uterine trouble, together with her anemic condition, continued, with more or less severity, till the 15th of August, 1854, when she was suddenly attacked, at midnight, with excessive difficulty of breathing, accompanied with a distinct croupy inspiration and cough.

She was largely bled — even to fainting, hot fomentations were made to the throat, and after nearly an hour of intense suffering from impending suffocation, the spasm of the glottis was relieved, and nothing remained of the difficulty save a slight croupy cough and the prostration consequent upon the bleeding.

Case II. Mrs. S., aged forty-two; married, is the mother of two children, the youngest eighteen years old. Soon after the birth of her last child — which was attended with no untoward symptoms — her recovery being good; she discovered a small tumor in the right iliac region. At first it was unattended with pain, but as it became larger it was accompanied with considerable pain of a lancinating character, which radiated from the tumor to different parts of the abdomen and down the thigh. It continued to enlarge, causing her to suffer more or less, till, when apparently at the acme of her distress, she was attacked with profuse uterine hæmorrhage.

At first the hæmorrahage was very severe — completely prostrating her, and bringing her rapidly into a state of anemia; but after several months it became less intense — recurring with severity at longer and longer intervals—until she considered herself quite free from any difficulty of that kind, so far had she recovered.

During the hæmorrahagic condition the ovarian tumor enlarged but little, if any; but as soon as that was moderated, it began, yet more slowly than before, to increase, but attended with less pain.

Matters continued in this manner till last October, when she came under my charge, with a return of the uterine hemorrhage. I had visited her but two or three times, and had first returned from a call at her residence, when I was summoned in haste to attend her again — the messenger telling me that she was "choking to death." I immediately repaired to her bed-side and found her propped up with pillows—the windows thrown open—her eyes projecting from their sockets and staring wildly—her respiration labored, and accompanied with a croupy cough, which came on in paroxysms, and was attended with the peculiar crowing inspiration of spurious croup.

I ordered hot fomentations to the throat, and discovering excessive tenderness over the spine in the cervical and upper dorsal regions, I made a pretty free appiication of the iodide of mercury oint. In addition to these measures, I administered chloroform by inhalation, and was happy to see, in a short time, that the severity of the attack had passed, and that she could breathe with but little difficulty. The harsh, croupy cough continued for a day or two.

The above cases, I consider interesting specimens of purely *reflex action*, and whose interest is heightened from occurring in adults. Laryngismus stridulus or spurious croup is a *very* rare affection in those of mature years, and probably would not have been induced in these patients, had not the spinal cord been rendered exceedingly sensitive from the long existing uterine difficulty.

